# Supplementary material for: Causal Relationship between Aging and Anorexia Nervosa: A White-Matter-Microstructure-Mediated Mendelian Randomization Analysis
Source: Biomedicines. 2024 Aug 16;12(8):1874. doi: 10.3390/biomedicines12081874 (PMC11351342; doi:10.3390/biomedicines12081874)
Supplement: Supplementary file 1 [file biomedicines-12-01874-s001.zip › Supplementary Figure/Figure S4/Singe SNP plot of MR analysis for TL to ubm-b-1724.pdf]

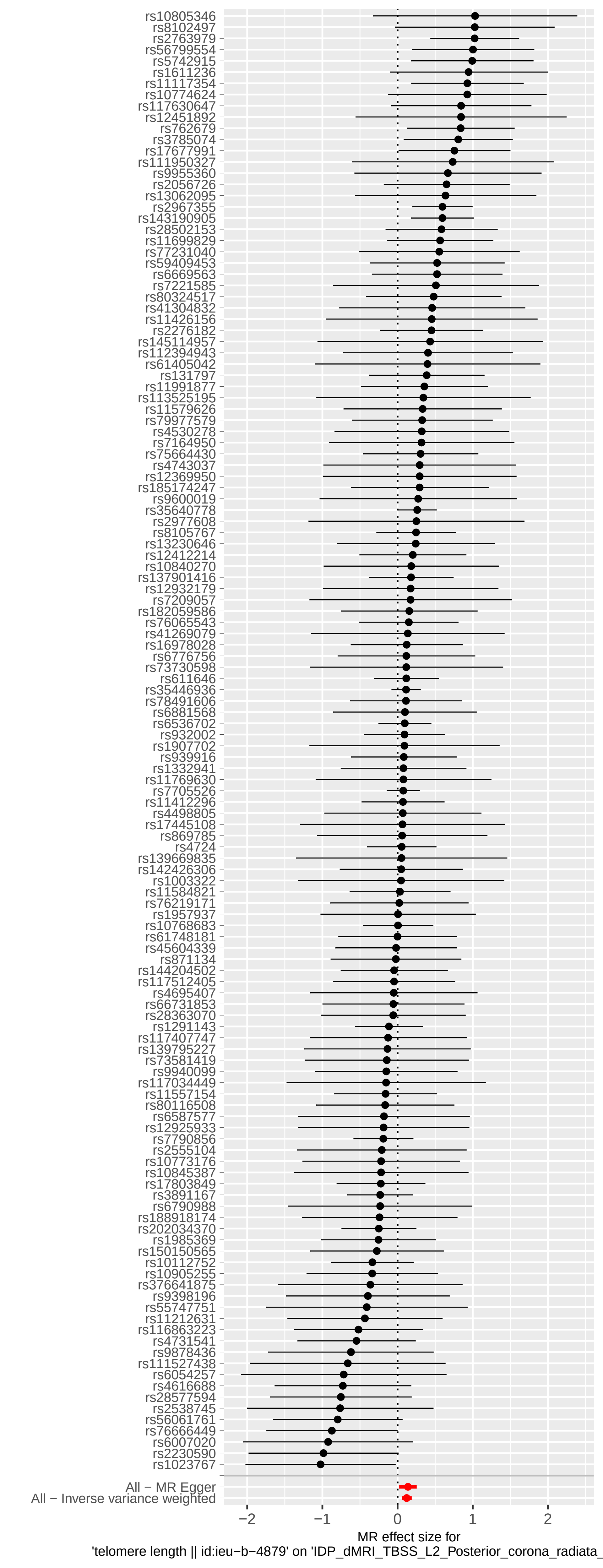
rs10805346
rs8102497
rs2763979
rs56799554
rs5742915
rs1611236
rs11117354
rs10774624
rs117630647
rs12451892
rs762679
rs3785074
rs17677991
rs111950327
rs9955360
rs2056726
rs13062095
rs2967355
rs143190905
rs28502153
rs11699829
rs77231040
rs59409453
rs6669563
rs7221585
rs80324517
rs41304832
rs11426156
rs2276182
rs145114957
rs112394943
rs61405042
rs131797
rs11991877
rs113525195
rs11579626
rs79977579
rs4530278
rs7164950
rs75664430
rs4743037
rs12369950
rs185174247
rs9600019
rs35640778
rs2977608
rs8105767
rs13230646
rs12412214
rs10840270
rs137901416
rs12932179
rs7209057
rs182059586
rs76065543
rs41269079
rs16978028
rs6776756
rs73730598
rs611646
rs35446936
rs78491606
rs6881568
rs6536702
rs932002
rs1907702
rs939916
rs1332941
rs11769630
rs7705526
rs11412296
rs4498805
rs17445108
rs869785
rs4724
rs139669835
rs142426306
rs1003322
rs11584821
rs76219171
rs1957937
rs10768683
rs61748181
rs45604339
rs871134
rs144204502
rs117512405
rs4695407
rs66731853
rs28363070
rs1291143
rs117407747
rs139795227
rs73581419
rs9940099
rs117034449
rs11557154
rs80116508
rs6587577
rs12925933
rs7790856
rs2555104
rs10773176
rs10845387
rs17803849
rs3891167
rs6790988
rs188918174
rs202034370
rs1985369
rs150150565
rs10112752
rs10905255
rs376641875
rs9398196
rs55747751
rs11212631
rs116863223
rs4731541
rs9878436
rs111527438
rs6054257
rs4616688
rs28577594
rs2538745
rs56061761
rs76666449
rs6007020
rs2230590
rs1023767
All − MR Egger
All − Inverse variance weighted
−2
−1
0
1
2
MR effect size for
'telomere length || id:ieu−b−4879' on 'IDP_dMRI_TBSS_L2_Posterior_corona_radiata_
